# *Lactobacillus fermentum* Stimulates Intestinal Secretion of Immunoglobulin A in an Individual-Specific Manner

**DOI:** 10.3390/foods11091229

**Published:** 2022-04-25

**Authors:** Liya Mei, Ying Chen, Jialiang Wang, Jian Lu, Jianxin Zhao, Hao Zhang, Gang Wang, Wei Chen

**Affiliations:** 1 State Key Laboratory of Food Science and Technology, Jiangnan University, Wuxi 214122, China; 6190112082@stu.jiangnan.edu.cn (L.M.); Manju_ying@163.com (Y.C.); wjl120587@126.com (J.W.); zhaojianxin@jiangnan.edu.cn (J.Z.); zhanghao61@jiangnan.edu.cn (H.Z.); chenwei66@jiangnan.edu.cn (W.C.); 2 School of Food Science and Technology, Jiangnan University, Wuxi 214122, China; 3 Department of Gastroenterology, Affiliated Wuxi No. 2 People’s Hospital of Nanjing Medical University, Wuxi 214122, China; 4 (Yangzhou) Institute of Food Biotechnology, Jiangnan University, Yangzhou 225004, China; 5 National Engineering Center of Functional Food, Jiangnan University, Wuxi 214122, China; 6 Wuxi Translational Medicine Research Center and Jiangsu Translational Medicine Research Institute Wuxi Branch, Wuxi 214122, China

**Keywords:** probiotics, Immunoglobulin A, immunity, gut microbiota, Immunoglobulin A-coated bacteria

## Abstract

Immunoglobulin A (IgA), as the most secreted immunoglobulin in the intestine, plays an irreplaceable role in mucosal immunity regulation. Previous studies have indicated that *Lactobacillus* showed strain specificity in stimulating the secretion of IgA through intestinal mucosal lymphocytes. The reason for this phenomenon is not clear. The current studies have been aimed at exploring the effect of a strain on the secretion of IgA in the host’s intestine, but the mechanism behind it has not been seriously studied. Based on this, we selected five strains of *Lactobacillus fermentum* isolated from different individuals to determine whether there are intraspecific differences in stimulating the secretion of IgA from the intestinal mucosa. It was found that IgA concentrations in different intestinal segments and faeces induced by *L. fermentum* were different. 12-1 and X6L1 strains increased the secretion of IgA by the intestine significantly. In addition, different strains of *L. fermentum* were also proven to have different effects on the host gut microbiota but no significant effects on IgA-coated microbiota. Besides, it was speculated that different strains of *L. fermentum* may act on different pathways to stimulate IgA in a non-inflammatory manner. By explaining the differences of IgA secretion in the host’s intestine tract stimulated by different strains of *L. fermentum*, it is expected to provide a theoretical basis for the stimulation of intestinal secretion of IgA by *Lactobacillus* and a new direction for exploring the relationship between *Lactobacillus* and human immunity.

## 1. Introduction

There are approximately 10^14^ microorganisms in the intestinal cavity, including bacteria, fungi, viruses and protozoa [1]. The gut microbiota promotes the development and response of the host mucosal immune system, enhances the close association between intestinal epithelial cells and antagonises pathogens [2]. The intestinal mucus layer is composed of mucin secreted by goblet cells, antimicrobial peptides secreted by intestinal epithelial cells and secretory immunoglobulin A (IgA) produced by B cells. IgA can effectively inhibit the adhesion and colonization of some bacteria in the intestinal epithelium [3].

The intestine is not only the site of food digestion and absorption but is also an important immune organ [4]. Through coevolution, the mammalian host and microorganism have formed a mutually beneficial symbiotic relationship. The host provides nutrition and a living environment for microorganisms, while microorganisms promote the steady-state balance of the intestine by participating in and regulating a series of physiological activities of the host [5]. Evidence suggests that a balance between intestinal bacteria and the mucosal immune system is an important factor in the development of intestinal mucosal inflammation [6,7]. The mucosal immune system elicits an effective defence against pathogens that invade the mucosa. Furthermore, proper tolerance needs to be developed against microbes that live in symbiosis with organisms in the mucosa [8].

IgA is the most commonly produced immunoglobulin in mammals [9]. The specific immune response in the gut is mainly through the production of IgA, IgM and IgG. Among them, IgA can inhibit the colonization and proliferation of some microorganisms in the mucus layer. Also, IgA can combine with some microorganisms to varying degrees to regulate the gut microbiota. There is no substitute for the role of IgA in intestinal immunity. At present, there have been many reports of probiotics that can regulate the level of IgA in the intestinal tract. It is known that *L. plantarum* YU could stimulate the secretion of IgA in Peyer’s patches by stimulating Th1 immune responses [10]. *Pediococcus acidilactici* K15 promoted the secretion of IgA in the oral mucosa, and this response was mainly induced via IL-10 [11]. *L. fermentum* CECT5716 enhanced IgA secretion in rats during pregnancy [12]. *L. fermentum* UCO-979C increased intestinal IgA levels while activating TLR4 [13]. The current studies mainly focus on how pathogenic bacteria stimulate host immunity and then affect the production of IgA. There is a lack of in-depth mechanism studies on how *Lactobacillus* promotes IgA secretion. In addition, it has been reported that the content of IgA in human faeces can be increased by diets containing high concentrations of acetic acid [14]. However, it is not known whether the SCFAs produced by the host’s gut microbiota are responsible for stimulating the host’s gut to secrete IgA. Most studies on the effect of *Lactobacillus* on intestinal IgA secretion remain on inter-species differences, and there are few studies on whether there are intra-species differences. In the previous study, we used forty strains of different species of *Lactobacillus* to conduct in vitro cell experiments and animal experiments (unpublished data). The results showed that among the various species of *Lactobacillus*, *L. fermentum* had the best potential for stimulating intestinal secretion of IgA. In this study, we selected five strains of *L. fermentum* derived from different human donors to investigate whether the hosts had differences in their corresponding response to IgA.

## 2. Materials and Methods

### 2.1. Bacterial Treatment

The five strains of *L. fermentum* (*L. fermentum* X6L1 [CCFM1225], *L. fermentum* 12-1 [CCFM1226], *L. fermentum* S24-1, *L. fermentum* 24M1 and *L. fermentum* 20M5) used in this study were isolated from faeces of healthy individuals and stored at the culture collection of food microorganisms in Jiangnan University (Wuxi, Jiangsu, China). The sources of these strains are shown in Table 1. The study did not involve human experiments, and the faecal samples containing the isolated strains were collected from healthy volunteers and did not cause any foreseeable risk or discomfort to the participants. The volunteers signed a written informed consent form or obtained the consent of their legal guardian. This study used previously isolated and preserved strains. Strain isolation was not involved in this study. All bacterial strains were cultured in modified de Man, Rogosa and Sharpe broth and incubated at 37 °C under anaerobic conditions (400TG; Electrotek, West Yorkshire, UK). Bacterial cells were collected by centrifugation at 6000× *g* for 5 min and suspended in a small volume of 30% glycerol solution to prepare a stock, and were stored at −80 °C for further treatment. The stock was diluted with sterilised phosphate-buffered saline (PBS) to yield a final concentration of 10^9^ colony-forming units (CFU)/mL for oral administration.

### 2.2. Animal Experiments

**Figure 1:**
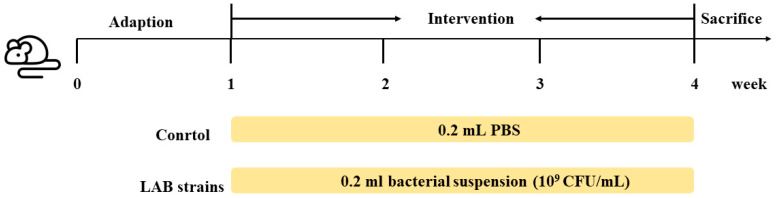
Schematic of the animal experiment. Five strains were included in the lactic acid bacteria (LAB) groups, *Lactobacillus* strains X6L1, S24-1, 12-1, 24M1 and 20M5.

The animal experiments involved in this study were carried out in the Animal Centre of Jiangnan University under environmentally controlled conditions (12-h light–dark cycle at 22 °C ± 3 °C and humidity of 55% ± 10%). All experiments were approved by the Animal Ethics Committee of Experimental Animal at Jiangnan University (JN. No:20210315c1080601 [012]). Three-week-old C57BL/6J female mice were purchased from the Model Animal Research Centre of Vital River (Shanghai, China). Standard food and sterile water were provided *ad libitum*. After acclimatisation for 7 days, 36 mice were randomly divided into six groups (*n* = 6/group). The control group was gavaged with 0.2 mL PBS solution. The X6L1, S24-1, 12-1, 20M5 and 24M1 groups were gavaged with 0.2 mL bacterial suspension (10^9^ CFU/mL) every day for three weeks. The experimental procedure timeline is shown in Figure 1. Mice were randomly assigned to the control group and different experimental condition groups using simple randomisation.

### 2.3. Tissue Processing

On the 7th, 14th and 21st days of the experiment, the faeces of mice were collected, placed on ice immediately and transferred to a freezer at −80 °C for storage. On the last day of the experiment, the mice were intraperitoneally injected with isoflurane after fasting for 12 h. One hour after intraperitoneal injection, the mice were sacrificed and their blood was collected. The blood was centrifuged at 2000× *g* for 15 min. The supernatant was collected as serum and stored in a freezer at −80 °C for later use. The mice were dissected, and the duodenum, jejunum, ileum, caecum, colon and other tissues were collected. Part of the intestinal tissues were stored in 40 g/L paraformaldehyde for later immunofluorescence staining. Of the remaining intestinal tissue, the caecal and colon contents were scraped, and the remaining tissue samples and caecal and colon contents were immediately placed into liquid nitrogen for rapid freezing. These samples were then stored at −80 °C for later use [15].

### 2.4. Real-Time Polymerase Chain Reaction

Approximately 20 mg of mouse colon that was preserved at −80 °C were placed together with high-temperature inactivated zirconia beads into an enzyme-free centrifuge tube. We added 1 mL of TRIzol and fully crushed the sample with a high-throughput crusher (SCIENTZ-48, Ningbo, China). The supernatant was collected, protein impurities and DNA were precipitated with chloroform and isopropanol was added for static precipitation. After discarding the supernatant, 75% pre-cooled ethanol was added to clean the extracted RNA twice, the solvent was left to air dry and the sample was re-dissolved with diethylpyrocarbonate-treated water. The purity and integrity of the extracted RNA were tested by measuring its optical density at 260/280 nm. In accordance with the instructions provided by the manufacturer of the Vazyme kit, complementary DNA was synthesised with the extracted total RNA as a template for real-time fluorescence quantitative polymerase chain reaction (qPCR) detection (Bio-Rad, Berkeley, CA, USA). The PCR system was prepared in accordance with the instructions of the qPCR mix, and the real-time qPCR program was run [16]. The transcription levels of genes encoding the polymeric immunoglobulin receptor (pIgR), myeloid differentiation factor 88 (MyD88), B-cell activating factor receptor (BAFFR), epidermal growth factor receptor (EGFR) and activation induced cytidine deaminase (ACIDA) in the mouse colon were measured by real-time qPCR. The primer sequences of mouse pIgR, MyD88, BAFFR, EGFR and ACIDA were found on the PrimerBank website (accessed on 19 January 2022, https://pga.mgh.harvard.edu/primerbank/index.html). Sonny Biotechnology Co., Ltd. (Shanghai, China) synthesised these primers. The specific primer information is shown in Table 2.

### 2.5. Determination of Short Chain Fatty Acids

Freeze-dried stool samples (50 mg) were homogenised in 500 μL of saturated NaCl solution and acidified with 40 μL of 10% sulphuric acid. Diethyl ether (1 mL) was added to the samples to extract short-chain fatty acids, after which the samples were centrifuged at 14,000× *g* for 15 min at 4 °C. Each supernatant (1 μL) was injected into an Rtx-WAX capillary column for gas chromatography–mass spectrometry analysis (QP2010 Ultra; Shimadzu, Kyoto, Japan). The initial oven temperature (100 °C) was increased to 140 °C at a rate of 7.5 °C min^−1^. The temperature was then further increased to 200 °C at a rate of 60 °C min^−1^ and maintained for 3 min. Helium was used as the carrier gas (flow rate: 0.89 mL min^−1^; column head pressure: 62.7 kPa). The injector temperature was set at 240 °C. The mass spectrometer was set at an ion source temperature of 220 °C, an interface temperature of 250 °C and a scan range of 2–100 *m*/*z* [17].

### 2.6. Enzyme-Linked Immunosorbent Assay

The faeces, duodenum, jejunum, ileum and colon, which were preserved at −80 °C, were thawed and the adipose tissue was removed. Pre-cooled sterile PBS solution was added according to a weight ratio of 1:9. The samples were then placed into a centrifuge tube together with cleaned and sterilised zirconia beads, broken down with a high-throughput crusher and then centrifuged at 4 °C for 2000× *g* for 10 min [18].

The supernatant was collected for assay measurement. Interleukin (IL)-1β, IL-6 and IL-17 concentrations were measured using mouse enzyme-linked immunosorbent assay kits according to the manufacturer’s protocol (R&D, Minneapolis, MN, USA). IgA and IgG concentrations were measured using mouse enzyme-linked immunosorbent assay kits according to the manufacturer’s protocol (Elabscience Biotechnology Co., Ltd., Wuhan, China). Protein was measured in intestinal tissue and faeces using the bicinchoninic acid assay (Beyotime Biotechnology, Shanghai, China).

### 2.7. Immunofluorescence of IgA in Plasma Cells in Mouse Intestinal Segments

After sacrificing the mice, approximately 1 cm of mouse intestinal tissue was carefully cut to avoid mechanical damage to the colon tissue as much as possible. After removing other tissues attached to the intestinal tissue, the residual faeces were removed by rinsing with pre-cooled normal saline, and then 4% paraformaldehyde solution was immediately added for fixation. The samples were embedded within 48 h: after washing the samples with clean water to remove excess fixative, they were successively treated with an ethanol gradient and xylene for dehydration and immersed in paraffin wax. The wax-soaked colon tissue was embedded using a Leica tissue embedding machine. After cooling, sections were cut with a hand wheel Leica tissue slicer. The sections were spread on slides and dried for subsequent staining [19]. The sections were stained overnight using the following reagents for immunofluorescence analysis: goat anti-mouse IgA alpha chain antibody (1:500, ab97231, Abcam, Shanghai, China), an FITC immunofluorescence detection kit (E670007, BBI, Shanghai, China) and 4′,6-diamidino-2-phenylindol dihydrochloride (Fcmacs, Nanjing, China).

### 2.8. Isolation and Identification of IgA-Coated Bacteria

IgA-coated bacteria were collected from faeces using magnetic bead-based enrichment. Briefly, faeces were suspended at 20% in pre-reduced PBS containing 0.5% Tween 20 (PBST) and protease inhibitors (1 mg/mL leupeptin, 1.6 mg/mL aprotinin; Sigma-Aldrich, St. Louis, MO, USA). The faeces were homogenised and centrifuged at 400× *g* to remove large debris. The supernatant was then centrifuged at 8000× *g* to pellet bacteria and washed with PBST three times. The bacterial pellet was resuspended in pre-reduced PBS supplemented with 0.25% bovine serum albumin, 5% goat serum and biotinylated goat anti-mouse IgA (ab97233, Abcam, Shanghai, China). After being washed, the biotinylated samples were mixed with streptavidin-coated magnetic beads to allow crosslinking (D110557; BBI, Shanghai, China). IgA-coated bacteria were separated from the suspension with the aid of a magnet. The collected bacteria were washed three times with PBST. DNA was quantified and pooled at equal concentrations by following the instructions of the Qubit dsDNA Assay Kit (Life Technologies, Carlsbad, CA, USA). All isolated strains were further typed by 16S rDNA high-throughput sequencing [20,21].

### 2.9. 16 S rDNA High-Throughput Sequencing

The total DNA of bacteria in fresh stool was extracted using the Fast DNA Stool Kit (MP Biomedicals, Carlsbad, CA, USA). PCR amplification of the 16S rDNA gene was performed using universal primers (341 forward: 5′-CCTAYGGGRBGCASCAG-3′ and 806 reverse: 5′-GGACTACNNGGGTATCTAAT-3′). The PCR products were purified using the TIANgel Mini Purification Kit (Tiangen, Beijing, China).

DNA was quantified and pooled at equal concentrations by following the instructions of the Qubit dsDNA Assay Kit (Life Technologies, Carlsbad, CA, USA). Samples were barcoded and finally paired-end sequenced on the Illumina MiSeq PE300 platform by following the manufacturer’s protocol. A gene sequencing analysis using 16S rDNA was performed with Quantitative Insights into Microbial Ecology version 2 (QIIME2) [22]. Amplicon sequence variants were rarefied to 10,000 according to the sampling depth.

The composition of *Lactobacillus* species was analysed and modified to run on QIIME2 [22]. The amplicon sequence variants were rarefied to 3000 according to the sampling depth. Subsequent processing was similar to that for 16S rDNA gene analysis unless otherwise noted.

### 2.10. Statistical Analysis

All data in this study are expressed as the mean ± standard deviation and were plotted with Prism 7 and Origin Pro 2021. We used one-way analysis of variance or Welch’s *t* test. The Spearman correlation coefficient was calculated. The results were corrected by False Discovery Rate (FDR) and with *p* less than 0.05 were retained. *p* < 0.05 indicated significance of the data (compared with the control group), and 95% confidence intervals are shown. CaseViewer was used to intercept the visual field of photographs after a slice scanner was used. Image J was used for immunofluorescence analysis, Xcalibur was used for gas chromatography–mass spectrometry off-machine data analysis and Origin Pro 2021 was used for correlation analysis. Statistical Analysis of Metagenomic (and other) Profiles (STAMP) was used for the Welch’s t test [23].

## 3. Results

### 3.1. L. fermentum Affects Intestinal Secretion of IgA

**Figure 2:**
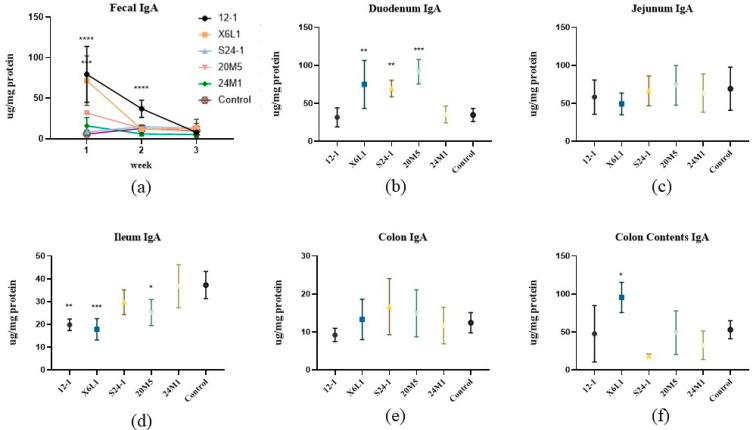
Effect of *L. fermentum* on IgA concentration in faeces and intestinal tract of normal mice (**a**) IgA in one to three week faeces; (**b**–**f**) Duodenum, jejunum, ileum, colon and colon contents were detected after three weeks by ELISA, “*” indicates *p* < 0.05, and “**” indicates *p* < 0.01, “***” indicates *p* < 0.001, “****” indicates *p* < 0.0001 (compared with control group).

After gavage for 1 week, compared with the control group, IgA concentrations in mouse faeces changed by varying degrees according to the *Lactobacillus* strain. The 12-1 and X6L1 strains significantly increased IgA concentrations in the faeces, but there were no significant changes in the S24-1, 20M5 or 24M1 groups (Figure 2a). During continuous gavage for three weeks, IgA concentrations in the faeces showed a downward trend and gradually approached those in the control group. IgA concentrations in each mouse intestinal segment were detected after three weeks of gavage. There were differences in IgA concentrations in the duodenum and ileum between the *Lactobacillus* strain groups (Figure 2b,d). IgA concentrations in the X6L1, S24-1 and 20M5 groups were significantly higher than those in the control group in the duodenum. However, IgA concentrations in the 12-1, X6L1 and 20M5 groups were significantly lower than those in the control group in the ileum. After gavage for three weeks, IgA concentrations were significantly higher in the X6L1 group than in the control group in the colonic contents, but this difference was not significant in the colon (Figure 2e).

**Figure 3:**
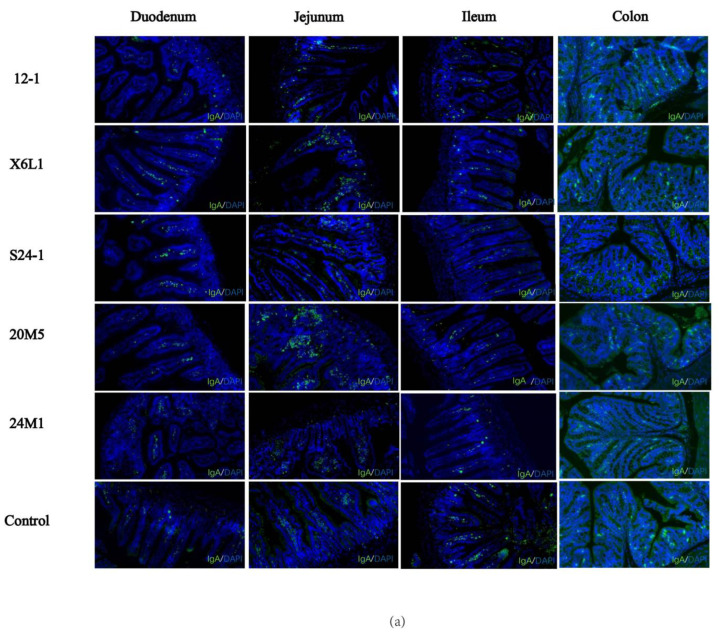

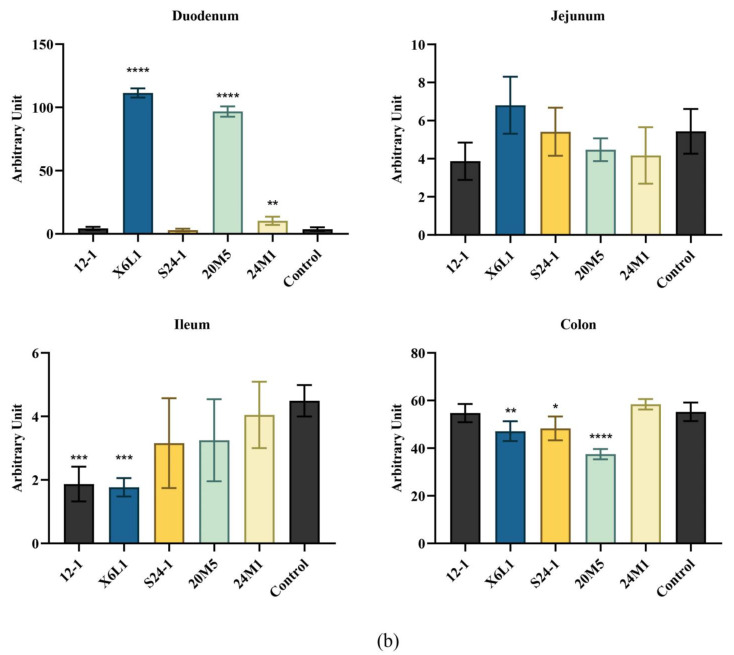
Effect of *L. fermentum* on IgA^+^ plasma cell in intestinal tract of normal mice.(**a**) The IgA immunofluorescence map of each intestinal segment of mice was obtained from the visual field of the photos intercepted by the CaseViewer (10×, green-IgA, blue-DAPI); (**b**) The average fluorescence intensity of immunofluorescence images of mouse intestinal tissues treated by Image J “*” indicates *p* < 0.05, and “**” indicates *p* < 0.01, “***” indicates *p* < 0.001, “****” indicates *p* < 0.0001 (compared with the control group).

An immunofluorescence assay on paraffin sections of mouse intestinal segments showed that IgA in plasma cells was sporadically distributed in the duodenum and colon, compared with the control group. IgA was also observed in the jejunum and ileum, but the number of plasma cells was low (Figure 3a,b). There was no difference in the fluorescence intensity of IgA and plasma cells in the jejunum. The IgA content in the intestinal segments was different among the groups and decreased with the direction of intestinal peristalsis. As shown in Figure 3b, compared with the control group, the fluorescence intensity of IgA in different intestinal segments of *Lactobacillus*-treated mice were different. The IgA plasma cell content in the duodenum was significantly higher than that in the other intestinal segments. X6L1 and 20M5 significantly increased the immunofluorescence intensity of IgA plasma cells in the duodenum. In the ileum, 12-1 and X6L1 decreased the fluorescence intensity. X6L1, S24-1, 20M5 also reduced the immunofluorescence intensity of IgA plasma cells in the colon.

### 3.2. L. fermentum Affects the Host Gut Microbiota and IgA-Coated Bacteria

**Figure 4:**
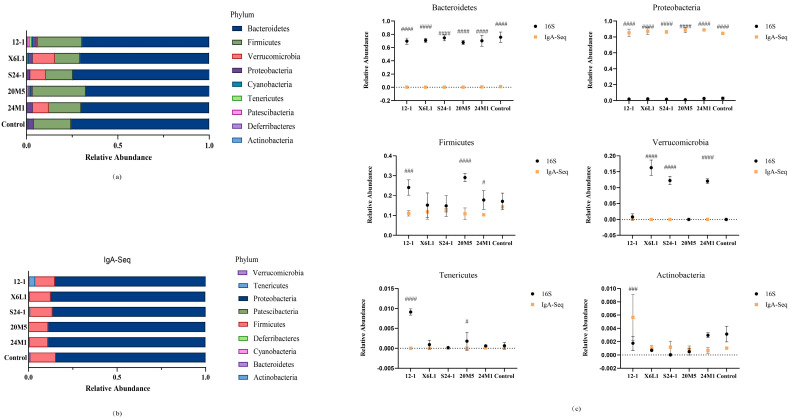
Effect of *L. fermentum* on gut microbiota in mice at phylum level. (**a**) Mouse colon contents 16S rDNA sequencing, phylum level relative abundance; (**b**) Mouse colon contents IgA−coated bacteria 16S rDNA sequencing, phylum level relative abundance; (**c**) Results of comparison between IgA−coated bacteria and colon contents microbiota on gate level (“#”indicates *p* < 0.05, “###” indicates *p* < 0.001, “####” indicates *p* < 0.0001 (compared with 16S)).

The microbiota of the mouse colon was examined by IgA immunomagnetic beads and 16S rDNA analysis. Firmicutes and Bacteroidetes comprised the main part of the microbiota on the whole. However, there were some differences in Actinomycetes, Tenericutes and Verrucous in the microbiota. Most IgA-binding bacteria were Proteobacteria and Firmicutes (Figure 4a,b). The overall changes in each group tended to be similar, but the proportion of IgA-bound Actinomycetes was significantly higher in the 12-1 group (Figure 4c).

**Figure 5:**
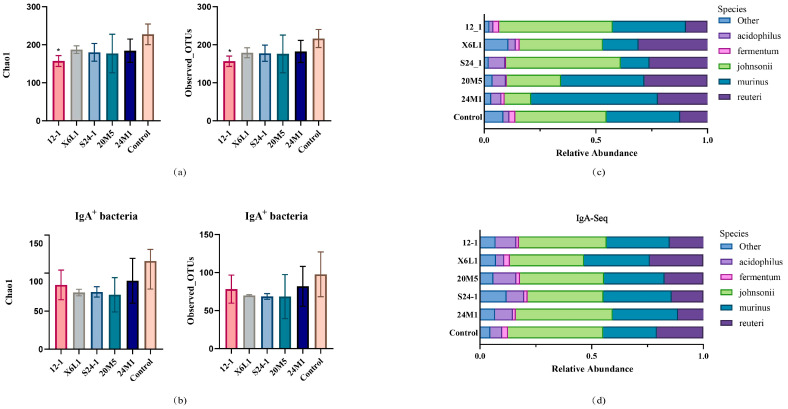
Effect of *L. fermentum* on gut microbiota in mice at α diversity and species level. (**a**) Mouse colon contents bacterial α-diversity calculated by Chao1 and observed OTUs; “*” indicates *p* < 0.05. (Compared with control group). (**b**) IgA-coated bacterial α−diversity calculated by Chao1 and observed OTUs; (**c**) 16S rDNA sequencing of mouse colon contents, relative abundance of *Lactobacillus* species level; (**d**) IgA−coated bacteria 16S rDNA sequencing of mouse colon contents, relative abundance of *Lactobacillus* species level.

The Chao1 index and operational taxonomic unit calculation and analysis showed that the 12-1 strain reduced the overall diversity of the host colon intestinal microbiota, but it did not affect the diversity of IgA-coated bacteria (Figure 5a,b). At the level of *Lactobacillus* species, the relative abundance of *L. johnsonii*, *L. reuteri*, *L. acidophilus*, *L. murinus* and *L. fermentum* was high. Different strains of *L. fermentum* had different effects on the host genus and species levels (Figure 5c,d).

**Figure 6:**
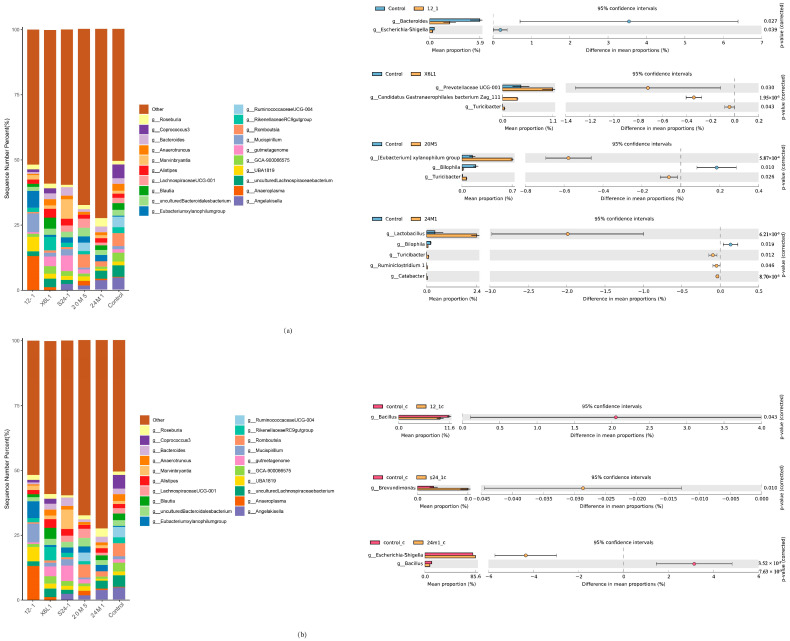
Effect of *L. fermentum* on gut microbiota in mice at genera level. (**a**) Relative abundance of bacteria in mouse colon and bacteria with differences by Welch’s t test at genus level; (**b**) Relative abundance of IgA-coated bacteria in mouse colon and bacteria with differences by Welch’s t test at genus level.

Welch’s t-test showed no significant difference in the S24-1 strain at the genus level compared with control group (data not shown; only groups with significant differences at the genus level are shown in Figure 6). Gavage with the 24M1 strain significantly increased the proportion of *Lactobacillus* species. Gavage with the 12-1 strain reduced the relative abundance of *Bacteroides*, *Escherichia* and *Shigella*. Gavage with the 20M5 strain increased the proportions of *Eubacterium xylanophilum* and *Turicibacter*, but decreased the proportion of *Bilophila*. Gavage with the X6L1 strain improved the relative abundance of *Prevotellaceae UCG-001*, *Candidatus gastranaerophilales bacteriom zag_ 1* and *Turicibacter*. (Figure 6a,b).

**Figure 7:**
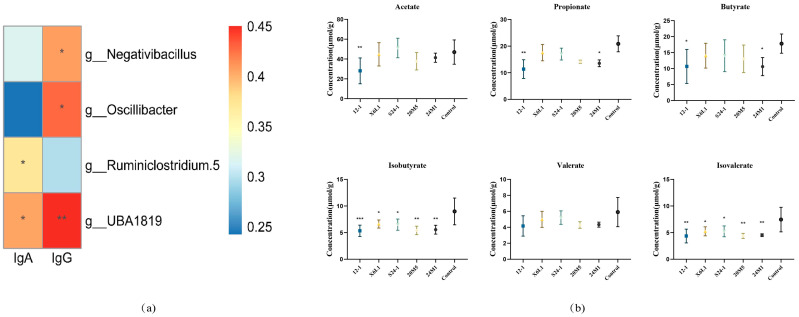
Analysis of *L. fermentum* on gut microbiota in mice and cecum SCFAs. (**a**) The correlation between IgA, IgG and colonic microbiota. “*” indicates *p* < 0.05, and “**” indicates *p* < 0.01 (compared with control group); (**b**) The content of short chain fatty acids in cecum. “*” indicates *p* < 0.05, and “**” indicates *p* < 0.01, “***” indicates *p* < 0.001 (compared with control group).

In the correlation analysis between the genus level and IgA, *Negativibacillus*, *Oscillibacter*, *Ruminiclostridium 5* and *UBA1819* were significantly correlated with IgA (Figure 7a). The proportions of *Escherichia* and *Shigella* in the 24M1 strain were higher and the abundance of *Bacillus* was lower than those in the control group. The 12-1 strain only reduced the abundance of *Bacillus*. There were no significant differences among the other species in the S24-1 strain except *Brevundimonas* increased. At the genus level, the X6L1 and 20M5 strains did not affect the relative abundance of IgA-bound bacteria.

All strains decreased the isobutyrate and isovalerate concentrations in the intestines. The 12-1 strain reduced the acetate, propionate and butyrate concentrations. The 24M1 strain also reduced the propionate and butyrate concentrations (Figure 7b).

### 3.3. L. fermentum May Stimulate Intestinal Production of IgA in Different Ways

**Figure 8:**
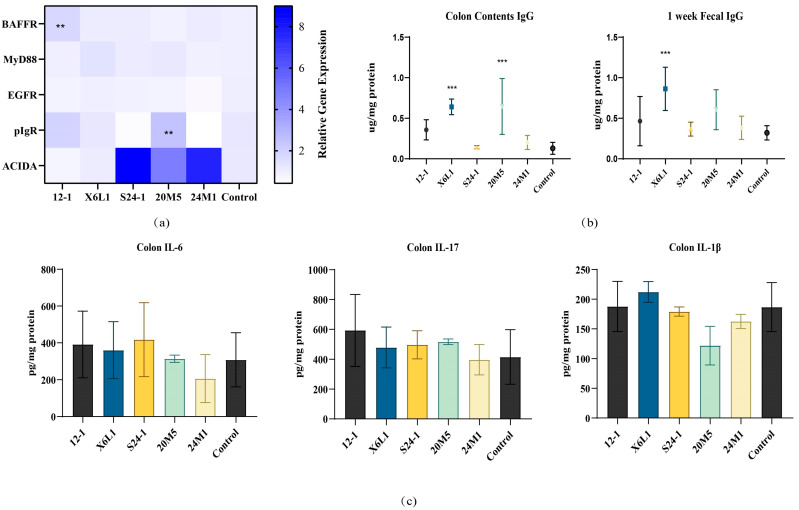
Effect of *L. fermentum* on IgA related protein gene expression and inflammatory factor in the colon of normal mice. (**a**) The relative gene expression of IgA related proteins, “**” indicates *p* < 0.01(compared with control group); (**b**) The content of IgG in feces and colonic contents in one week, “***” indicates *p* < 0.001 (compared with control group); (**c**) The content of colitis indicators.

The expression of genes encoding several reported IgA-related proteins in the mouse colon is shown in Figure 8. Of the two strains of *L. fermentum*, 12-1 and X6L1, that can increase the IgA content in faeces, only 12-1 upregulated BAFFR expression in the colon. The 20M5 strain upregulated pIgR expression, but the IgA concentration did not significantly change. There was no difference in gene expression related to IgA antibody class conversion among the five strains of *Lactobacillus* (Figure 8a).

### 3.4. L. fermentum May Produce IgA in a Non-Inflammatory Manner

To examine the effects of different strains of *L. fermentum* on IgA secretion from intestinal mucosa, we measured IgG concentrations in the faeces after gavage of one week and colon contents after gavage of three weeks, when IgA concentrations were high. Only the X6L1 strain increased IgG concentrations (Figure 8b). There were no differences in IL-6, IL-17 or IL-1β concentrations among the strains (Figure 8c).

## 4. Discussion

Host intestinal mucosal immunity is related to intestinal symbiotic bacteria [24]. IgA is the most secreted immunoglobulin in the intestine. However, there have been few studies on the mechanism by which common bacteria, especially lactic acid bacteria, affect host intestinal IgA [25]. Three problems need to be solved to determine how an antibody reaction is related to common microorganisms in the intestine. First, whether the IgA response is specific to the symbiotic bacteria that stimulate it is unknown. Second, how the IgA reaction adapts to the current bacteria in the intestine is unclear. Third, whether all bacteria can effectively induce IgA in an equal manner is unknown [13,26,27].

In this study, we used five strains of *L. fermentum* from different sources to examine whether there are intraspecific differences in the effect of inducing IgA. We found that not all *L. fermentum* strains stimulated IgA production in the gut. The increase in free IgA content in faeces may be due to (1) an increase in IgA plasma cells (2) and an increase in IgA transported from the intestinal lamina propria to the intestinal cavity. With regard to an increase in the free IgA content in faeces, *Lactobacillus* may promote the proliferation of B cells or stimulate antibody class switch recombination (CSR) in the B cells. Either of these possibilities could increase the number of IgA plasma cells at the corresponding effector site in mice. We performed a series of experiments to investigate this possibility. As shown in Figure 2 and Figure 3, compared with the control group, the concentrations of IgA in different intestinal segments of *Lactobacillus*-treated mice were different, suggesting that different *L. fermentum* strains had different effector sites in the intestine of mice. Immunofluorescence staining showed that the duodenum and colon had the most plasma cells. There were differences in the duodenum and ileum. Considering the results of IgA content in various intestinal segments in Figure 2b–f, it is speculated that X6L1, S24-1, and 20M5 first induced IgA-specific immunity in the duodenum and stimulated the differentiation of IgA plasma cells in the lamina propria. However, there was no difference in the fluorescence intensity of IgA plasma cells in the jejunum, so the effect sites of X6L1 and 12-1 may not be in the jejunum. The fluorescence intensity of X6L1 and 12-1 in the ileum were significantly lower than that of the control group and other *Lactobacillus* intervention groups. In addition, the ileum was the site with the lowest IgA content in both groups. X6L1, S24-1, and 20M5 decreased the fluorescence intensity of IgA plasma cells in the colon, while there was no difference in IgA concentration in colon tissue. However, from the content of IgA in colonic contents, it was significantly increased in the X6L1 group, significantly decreased in S24-1 group, and not significantly changed in the 20M5 group. This further indicates that different strains of *L. fermentum* have different effects on intestinal stimulating IgA. In the faeces, the IgA levels of the 12-1, X6L1 group were significantly higher than that of the control group, which means that the two groups of *L. fermentum* increased the content of IgA from the overall level of the intestinal tract. Notably, as shown in Figure 3b, 12-1 did not increase the number of IgA plasma cells in the gut. Therefore, it is speculated that 12-1 might alter the transport efficiency of IgA from the lamina propria, rather than promote the proliferation of IgA-producing cells. This indicated that different strains of *Lactobacillus* of the same species had differences in the way of stimulating the intestinal secretion of IgA. The small intestine may play an important role in the production of IgA.

In Figure 2a, during continuous gavage for 3 weeks, IgA concentrations in the faeces showed a downward trend and gradually approached those in the control group. With extended contact time between the intestinal mucosa and bacteria, IgA concentrations could not be maintained at a high level. Whether the immunogenicity of lactic acid bacteria is related to this issue is unclear [28]. After a period of adaptation in this study, the gut microbiota reached a new balance. The mucosal immunity developed tolerance to the experimental strains and no longer secreted too much IgA [29]. As a result, IgA concentrations in the faeces decreased with an increase in the experimental duration.

Considering an increase in IgA transported from the intestinal lamina propria to the intestinal cavity, *Lactobacillus* may stimulate B cell associated pathways protein gene expression in mouse intestinal epithelial cells [30]. This stimulation could result in a higher rate of transport of IgA produced in the lamina propria out of the intestinal cavity, which could improve the transport efficiency [31]. BAFF is involved in B cell homeostasis and survival including promoting B cell to plasma cell transformation, making it an important pathway for regulating CSR and antibody production [32]. As a BAFF receptor, the content of BAFFR directly reflects the number of plasma cells [32]. In Figure 8a, the up-regulation of BAFFR levels suggested that *L. fermentum* 12-1 may increase the number of plasma cells by stimulating colonic secretion of BAFF. As a transmembrane receptor, EGFR can widely regulate cell proliferation, differentiation, migration and survival, and participate in various physiological and biochemical reactions [33]. However, *L. fermentum* treatments showed no effect on EGFR levels. It is suggested that this species of *Lactobacillus* does not activate the EGFR pathway. MyD88 acts as a linker molecule in the TLR pathway. It is involved in mediating the activation of the NF-κB pathway, stimulating the secretion of cytokines, and transmitting inflammatory signals [34]. In this study, the gene transcription level of this protein in the colon did not change, which indicated that *L. fermentum* may not stimulate colon inflammation. pIgR located on the basal surface of intestinal epithelial cells can bind to IgA in the lamina propria, allowing it to pass through the epithelial cells to the mucosal surface to exert its protective effect [35]. Only the expression of pIgR was up-regulated in the 20M5 group, but there was no significant change in the concentration of IgA. It is speculated that other subclasses of antibodies such as IgG (Figure 8b) may be transported from the lamina propria to the intestinal lumen. As a subclass of immunoglobulin, IgA needs to complete complex biochemical reactions through a special antibody CSR in order to exert its corresponding efficacy. Antigens are transported to B cells through antigen-presenting cells [36]. After B cells are stimulated, activation-induced cytidine deaminase (AID) is activated accordingly and begins to transcribe specific gene regions on the immunoglobulin heavy chain [36]. The expression level of ACIDA is an important indicator to detect whether antibody CSR occurs [36]. The ACIDA transcription levels were up-regulated in the colon tissue of mice treated with S24-1, 24M1 and 20M5. But the data showed that these three strains did not up-regulate the level of IgA in the intestine. Therefore, it is presumed that B cells at this site undergo antibody CSR but have not matured into IgA^+^ plasma cells. They may be transformed into plasma cells that secrete other subclasses of antibodies such as IgG. From this, it is not difficult to infer that *Lactobacillus* that affects different levels of intestinal secretion of IgA may cause IgA-specific immunity in different ways. These pathways are not limited to BAFFR, MyD88, EGFR, pIgR, or ACIDA-related pathways.

An increase in IgA concentrations in the faeces may not only be the result of the direct action of *L. fermentum* but also could be due to a new balance of the intestinal microbiota. The relative abundance of *Lactobacillus* and *L. fermentum* did not increase at the genus or species level in this study (Figure 6a,b). However, *L. fermentum* affected the relative abundance of other members of the intestinal microbiota. The change in IgA concentrations in the intestine is a dynamic and comprehensive result. To further study the effect of stimulation of a single bacterium on host intestinal IgA, germ-free mice need to be colonised with single species of bacteria.

IgA plays different roles by binding to different bacteria [29]. IgA combines with pathogenic bacteria to achieve the effect of immune exclusion, and with symbiotic bacteria to help colonisation [22,25,26]. However, how IgA identifies pathogenic bacteria and symbiotic bacteria is unknown. Using the immunomagnetic beads combined with 16S rDNA analysis in this study, *L. fermentum* was showed to have different effects on the host colonic microbiota and IgA-coated bacteria. There were differences in *Escherichia*, *Shigella*, *Bacillus* and *Brevundimonas* at the genus level (Figure 6a,b). The effect of IgA on these genera may be different [27]. IgA can specifically target lipopolysaccharide in *Escherichia* and *Shigella* species to limit it to the intestinal mucus layer and reduce intestinal inflammation [35]. However, IgA induced by *Bacteroides fragilis* helps the bacterium cluster and anchor on the surface of the intestinal epithelium, thus providing it with a competitive advantage [28]. In this study, differences in IgA-coated bacteria were not related to IgA concentrations. It is doubtful whether the difference of microbiota caused by different *L. fermentum* has any effect on IgA concentration. At present, whether this result is a two-way effect caused by IgA and the gut microbiota is unclear. Exploring the causes of this phenomenon will require more in-depth research.

In recent years, there has been a debate on whether short-chain fatty acids are related to IgA. As a metabolite of gut microbiota, acetic acid has been formally known to stimulate the secretion of IgA [37]. *L. fermentum* is a heterologous fermentation strain which produces lactic acid and large amounts of acetic acid, ethanol and CO_2_ after fermentation [37]. However, acetic acid concentrations in the caecum did not increase in this study (Figure 7b). There was no correlation between IgA and short-chain fatty acids in the caecum. Short-chain fatty acids are mainly produced in the large intestine [38]. Indeed, IgA has been confirmed in the existing studies to be produced in the small intestine and enriched in the colon [38]. However, this conclusion is mainly proposed for pathogenic bacteria or invasive and strong bacteria that can cause IgA-T cell-dependent-PPs pathways, such as *Segmented filamentous bacteria* [4]. Whether all the bacteria only stimulate the small intestine to secrete IgA is unknown. Therefore, the causal relationship between IgA and short-chain fatty acids cannot be determined. Short-chain fatty acids concentrations in the small intestine might be strongly correlated with IgA concentrations. Initially, we would like to explore the effector site of *L. fermentum* to stimulate the intestinal secretion of IgA. It was not clear from the previous experiments that *L. fermentum* would stimulate IgA secretion in the small intestine, since the bacteria is mainly concentrated in the colon. Therefore, we detected the IgA-coated bacteria in the colon in order to explore the effect of *L. fermentum* on the gut microbiota. The path through which the 12-1 and X6L1 strains stimulate intestinal lymphocytes to secrete IgA will be the focus of a future study. However, we speculate that these two strains affect different reaction pathways to a great extent.

Intestinal IgA may provide immune protection and rejection in a non-inflammatory manner, which promotes the establishment of the host microbial interaction mechanism [39]. Interleukins are a class of cytokines that are produced by cells and have direct or indirect stimulatory effects on immune cells [39]. It plays a special role in innate and adaptive immunity. Macrophages, Th2 cells, vascular endothelial cells, and fibroblasts are the main sources of interleukins [39]. IL-6 can stimulate the proliferation and differentiation of B cells, and regulate the inflammatory response by releasing antibodies [39]. IL-17 is an early initiator of T cell-induced inflammatory response and can release pro-inflammatory cytokines to amplify the inflammatory response [40]. IL-1β is a cytokine and regulator secreted by macrophages to activate cellular immunity [40]. However, the contents of IL-6, IL-17 and IL-1β in the colon of all *L. fermentum* treated mice did not change significantly (Figure 8c). X6L1 and 12-1, which induced IgA, might affect the intrinsic and specific immunity of intestinal mucosa through their special epitopes or secondary metabolites, and then promote the secretion of IgA. Intestinal IgA might provide immune protection for the host in a non-inflammatory manner, promoting the interaction between the host’s gut microbiota and mucosal immunity, and maintaining immune balance. The cell walls of different strains of *L. fermentum* need to be investigated in further studies to determine whether the difference in antigenic determinants in the cell wall lead to a difference in the IgA response [41], whether different surface antigens stimulate IgA in the same manner and whether other types of lactic acid bacteria also have intraspecific differences in stimulating the secretion of IgA from the intestinal mucosa [41]. At present, the two-way effect between symbiotic bacteria and IgA is not well understood. IgA may have an undiscovered response mechanism in humoral immunity.

Notably, among the five tested strains of *L. fermentum*, two were from infants and three were from older people (Table 1). The strains that increased IgA concentrations in faeces were from the infants. Whether the bacteria that cause changes in IgA in the intestine are related to age and the physical condition of the provider are unknown [42]. An infant’s intestinal environment might be different from that of an adult, which may lead to differences in genes of the same bacteria and thus to different characteristics of the bacteria [42]. The intestinal microbiota and immune system in infants are in a process of development [43]. During this process, the production of IgA may be different from that in adults, which is stimulated by a stable intestinal microbiota [43]. Additionally, the induction of IgA by bacteria in different intestinal environments may be different between infants and adults. This difference may be at the levels of the gut microbiota and of different genotypes and physiological characteristics of the same species. This possibility would indicate a complicated interaction between the host gut microbiota and IgA [44]. There have been no relevant reports on whether the age or sex of the provider affects their intestinal IgA concentration. Therefore, this lack of knowledge could point to a new direction of IgA research.

## 5. Conclusions

Different strains of *L. fermentum* show differences in stimulating the secretion of IgA in the host intestine. Some of these strains increase the number of IgA plasma cells in the duodenum, which increases IgA concentrations, while some stimulate BAFFR expression to induce high IgA concentrations. Furthermore, IgA concentrations are not significantly correlated with short-chain fatty acid concentrations in the caecum. *L. fermentum,* from different sources, has different effects on the host intestinal microbiota, but it does not affect the diversity of IgA-coated microbiota. Additionally, *L. fermentum* may stimulate intestinal secretion of IgA through different reaction pathways in a non-inflammatory manner.

## Figures and Tables

**Table 1:** Strains used in this study.

Strain	Species	Origin	Age
12-1	*Lactobacillus fermenti*	Human feces, male	0
X6L1	*Lactobacillus fermenti*	Human feces, female	0
S24-1	*Lactobacillus fermenti*	Human feces, female	100
20M5	*Lactobacillus fermenti*	Human feces, male	83
24M1	*Lactobacillus fermenti*	Human feces, female	75

**Table 2:** Primer sequence.

Gene	Primer (5′-3′)
*pIgR*	F-AGGCAATGACAACATGGGG
	R-ATGTCAGCTTCCTCCTTGG
*MyD88*	F-CACCTGTGTCTGGTCCATTG
	R-CTGTTGGACACCTGGAGACA
*BAFFR*	F-GAAACTGCGTGTCCTGTGAG
	R-CTGAGGCTGCAGAGCTGTC
*EGFR*	F-GCCATCTGGGCCAAAGATACC
	R-GTCTTCGCATGAATAGGCCAAT
*ACIDA*	F-CGTGGTGAAGAGGAGAGATAGTG
	R-CAGTCTGAGATGTAGCGTAGGAA
*GAPDH*	F-TGTGTCCGTCGTGGATCTGA
	R-CCTGCTTCACCACCTTCTTGAT
